# Supplementary material for: In vivo tracking of transplanted macrophages with near infrared fluorescent dye reveals temporal distribution and specific homing in the liver that can be perturbed by clodronate liposomes
Source: PLoS One. 2020 Dec 10;15(12):e0242488. doi: 10.1371/journal.pone.0242488 (PMC7728253; doi:10.1371/journal.pone.0242488)
Supplement: Attachment — Submitted filename: response to comments 11-26.rtf [file pone.0242488.s001.rtf]

Our responses to each of the comments are as follows.  The modifications to the manuscript are tracked in yellow.

1) It appears that your ORCiD iD has not been validated in your Editorial Manager account and we are unable to proceed until that step is complete. 

	I made an ORCID iD newly and added its information.

2) "This study was carried out in strict accordance with the recommendations in the Guide for the Care and Use of Laboratory Animals of the National Institutes of Health. The protocol was approved by the Committee on the Ethics of Animal Experiments of the Nagoya University (Protocol Number: 24298). All procedures was performed under sodium pentobarbital anesthesia, and all efforts were made to minimize suffering.". To help ensure that the wording of your manuscript is suitable for publication, would you please also add this statement at the beginning of the Methods section of your manuscript file.
	We have added this statement in M&M.

3) Thank you for updating your data availability statement. You note that your data are available within the Supporting Information files, but no such files have been included with your submission. At this time we ask that you please upload your minimal data set as a Supporting Information file, or to a public repository such as Figshare or Dryad. 

	We have added the details of observation about the morphology and viability of macrophages in the revised manuscript insted of uploading supplementary data, tracked in yellow (page 9, line 144-146).
