# Supplementary material for: In vivo tracking of transplanted macrophages with near infrared fluorescent dye reveals temporal distribution and specific homing in the liver that can be perturbed by clodronate liposomes
Source: PLoS One. 2020 Dec 10;15(12):e0242488. doi: 10.1371/journal.pone.0242488 (PMC7728253; doi:10.1371/journal.pone.0242488)
Supplement: Attachment — Submitted filename: Response to reviewers.rtf [file pone.0242488.s002.rtf]

Response to the reviewers

Academic Editor: The use of liposomal clodronate is not sufficiently motivated in the text. For this reason, Authors must extensively clarify in their manuscript why they studied liposomal clodronate rather than other types of liposomal products.

As the academic editor suggested, we have added descriptions about advantages of liposomal clodronate to deplete macrophages (page 3, line56-57 and page 4, line 62-63).

Reviewer #1: The authors report the localization of fluorescent labelled macrophages within the lungs, spleen and liver and the impact of liposomal clodronate upon maintenance of fluorescence. They demonstrated that labelled macs accumulated fluorescence in the above organs, but not kidney or skin. Clodronate is bisphosphonate with a liposome and action is not mentioned within the article.

We are grateful for the useful comments and suggestions that have helped us to improve our paper. We have taken all these comments and suggestions into account in the revised version of our manuscript. 

1.	thioglycolate recruited macrophages are differentially activated through the recruitment process. The old literature (80s) spoke about the type of thioglycolate used and differential ability to phagocytose bacterial. The authors didn't mention the impact/activation of the type of recruitment upon subsequent homing of cells to the various locations. Some mention of cell surface receptors for endothelial or tissue ligands may be useful. Some of the differences are discussed in old literature, again such as J Immunol November 1, 2003, 171 (9) 4816-4823

As the reviewer suggested, we have added the description about difference of homing patterns between thioglycolate-recruited macrophages and macrophages established by other means  (page 14, line 232-234 and page 15, line 247).

2.	There should be discussion of liposomal clodronate and the induction of apoptosis. It is not mentioned.
As the reviewer suggested, we have added the description about apoptotic cell death of macrophages induced by clodronate (page 14, line 232-234 and page 15, line 247). 

3.	there is not discussion of statistical analysis of the findings.
As the reviewer suggested, we have added descriptions and asteriks which denotes statistical significance in the Material and Method and Results sections, and in the Figure 2, respectively.

4. the authors keep discussing the possibility of liposomal steroids, but did not study it. Liposomal clodronate has a putative role in autoimmune and transplant related conditions where on may want to remove tissue macrophage damage. It was not mentioned within the context of this manuscript. the fact that inert liposomes did not appear to alter macrophages and clodronate loaded liposomes was associated with loss of macrophage associated fluorescence suggests that this may be a way to gain targeted drug delivery, but it is very round about.

We are very sorry for the errors we made in the page 15, line 238 . Liposomal steroid should have been liposomal clodronate. We have corrected the errors. Additionally, we have changed the description about clodronate (page 3, line 56-58) since it was confusing and roundabout as the reviewer pointed out.


Reviewer #2: Nakayama et al presents a new method to track macrophages in vivo using bioluminescence and probe the method of depletion using clodronate liposomes.
Actually, the article checks a macrophage tracking method in vivo. Regarding the methodology used, the necessary controls are considered to validate the technique and the materials and methods are adequate to verify that the method works correctly. Perhaps it would be interesting to test it on immunodeficient mice or another animal species to see the possibilities of the technique. The Editor should assess whether it is of sufficient scientific quality to be published on Plos One.

We thank the reviewer for helpful suggestions to increase the quality of our manuscript. 
The reviewer pointed out that we should have used appropriate controls in the tracking experiments.  It is very difficult to pick up "appropriate controls". However, we have tracked down the fluorescence -labeled mesenchymal stromal cells in vivo after intravenous injection, showing that mesenchymal stromal cells transiently lodged in the lung and then moved to liver and spleen (now preparing for submission). The in vivo behavior of macrophages apparently differs from that of mesenchymal stromal cells. Thus, we concluded that the strong affinities for the lung, liver and spleen were specific to macrophages.  
The reviewer also proposed that we should test it on immunodeficient mice or another animal species to see the possibilities of the technique. We had similar ideas as well, but we gave up to perform experiments since chemokine systems are different between mice and humans (e.g. mouse CCL2 is 58% identical to human CCL2). Thus, we were afraid that we might not obtain accurate data when human cells were injected into immunodeficient mice. Additionally, we don't have an image analyzer for large animals
