# Supplementary material for: In vivo tracking of transplanted macrophages with near infrared fluorescent dye reveals temporal distribution and specific homing in the liver that can be perturbed by clodronate liposomes
Source: PLoS One. 2020 Dec 10;15(12):e0242488. doi: 10.1371/journal.pone.0242488 (PMC7728253; doi:10.1371/journal.pone.0242488)
Supplement: Attachment — Submitted filename: Response to Reviewers.docx [file pone.0242488.s003.docx]

Our responses to each of the comments are as follows. The modifications to the manuscript are tracked in yellow.

Reviewer #3: The paper by Nakayama and colleagues aim to image the biodistribution of Raw macrophages and see the effect of Clodronate liposomes on their depletion using optical imaging.

*Query: Since the authors use the XenolighDIR fluorescent dye they are imaging near infrared fluorescence and not bioluminescence so the title is misleading.*

Answer: As pointed out by the Reviewer 3, the term “*bioluminescence*” was inappropriate. Thus, we changed the title as “In vivo tracking of transplanted macrophages with near infrared fluorescent dye reveals temporal distribution and specific homing in the liver that can be perturbed by clodronate liposomes”.

*Query: Moreover since the perform direct labelling and the dye bound to cell membranes, there is no proof that cells retain it after 24h so for their analysis at 168h the authors should provide more evidence that they are looking at macrophages in the different organs and not at the free dye.*

Answer: The product sheet of Xenolight DIR tells that the two long 18-carbon chains insert into the cell membrane, resulting in specific and stable cell staining with negligible dye transfer between cells (https://www.perkinelmer.com/product/dir-125964).

There was virtually no free dye in the cell suspension, because we washed macrophages labeled with Xenolight DIR three times with PBS before we injected as reported previously [J Nucl Med 2009; 50: 1676-1682]. It has been reported that primary macrophages retained the Xenoligh DIR fluorescent dye over 7 days [J Nucl Med 2009; 50: 1676-1682].

In Fig2, fluorescence signal intensities were much decreased in the liver, spleen and lung of mice treated with Lipo-CL2MDP, where the number of macrophages was much decreased as well, suggesting that Xenolight DIR inserted into the cell membrane of macrophages were not trasferred to other cells.

We had wanted to identify cells with fluorescence as macrophages, but it was practically impossible because (1) the resolution of in vivo imaging system at our facility was too low to detect a single cell and (2) microscopic images of organs, immunostained with F4/80, could not be overlaid with fluorescence images obtained from in vivo imaging.

*Query: Moreover the paper lacks quantitative analysis. The author should report the fluorescence signal in the organ in relation to weight of the organ so that a comparison of the prevalence of the cells can be claimed and statistical analysis performed.*

Answer:

The intensities of fluorescence signal were expressed as photons/sec/unit area, not total photons of each organ. Thus, thickness, not weight, of organs may correlates with signal intensities. However, there is little and ignorable difference of thickness among organs, except for femors.

Each organ has unique structure (e.g. bones in the femurs, alveolars in the lung, and blood in the heart), which can attenuate the signal intensities to some extent. Statisitical analysis can not be performed among subgroups in different backgrounds. Thus, these were reasons why we analyzed trends, not statistical significances, in the macrophage accumulation as shown in Figure 1C and 2C. On the other hand, we performed statistical analysis between mice treated with clodronate liposomes and mice treated without clodronate liposomes to confirm the effect of clodronate liposomes on the macrophage accumulation since both subgroups had similar backgrounds.

*Query: Finally A. Taylor and colleagues ( doi: 10.1155/2018/2514796) injected bioluminescent RAW macrophages in animals and, although they also see signals in the liver , they were able to see it in the brain area too. So it would be nice to add this organ to the analysis.*

Answer: We did not analyze the intensity of fluorescence signal in the brain because minimal signal intensity was observed in the head region as shown Figure 1A after primary macrophages were administrated via tail vein. In the Contrast Media & Molecular Imaging paper (doi.org/10.1155/2018/2514796), the authors injected the luciferase gene-transduced RAW 264.7 cells via the left cardiac ventricle, suggesting that RAW 264.7 cells were directly delivered to the brain via large arteries. RAW 264.7 is a macrophage-like leukemia cell transformed by virus (<https://www.atcc.org/products/all/tib-71.aspx>). Thus, we believe that the route of administration and the cell type are likely cause of failure for us to detect the signal in the head.

We hope that the Editor finds that our re-revised manuscript adequately addresses the comments made by the reviewer #3 and that the manuscript is now ready for publication in the PLOS One.

Sincerely,

Takayuki Nakayama M.D., Ph.D.
Department of Transfusion Medicine, Aichi Medical University, 
1-1 Karimata, Yazako, Nagakute, Aichi 480-1195, Japan 
e-mail: tnaka@aichi-med-u.ac.jp
